# Particulate matter air pollution as a cause of lung cancer: epidemiological and experimental evidence

**DOI:** 10.1038/s41416-025-02999-2

**Published:** 2025-04-04

**Authors:** Meng Wang, Richard Y. Kim, Maija R. J. Kohonen-Corish, Hui Chen, Chantal Donovan, Brian G. Oliver

**Affiliations:** 1 School of Life Sciences, Faculty of Science, University of Technology Sydney, Ultimo, NSW Australia; 2 Woolcock Institute of Medical Research, Macquarie University, Sydney, NSW Australia; 3 Immune Health Research Program, Hunter Medical Research Institute and University of Newcastle, Newcastle, Australia; 4 Macquarie Medical School, Macquarie University, Sydney, NSW Australia; 5 Sydney Local Health District, Sydney, NSW Australia; 6 School of Medicine, Western Sydney University, Sydney, NSW Australia

**Keywords:** Lung cancer, Cancer models, Lung cancer

## Abstract

Air pollution has a significant global impact on human health. Epidemiological evidence strongly suggests that airborne particulate matter (PM), the dust components of polluted air, is associated with increased incidence and mortality of lung cancer. PM2.5 (PM less than 2.5 µm) from various sources carries different toxic substances, such as sulfates, organic compounds, polycyclic aromatic hydrocarbons, and heavy metals, which are considered major carcinogens that increase lung cancer risk. The incidence and mortality of lung cancer caused by PM2.5 exposure may be due to significant geographical differences, and can be influenced by various factors, including local sources of air pollution, socioeconomic conditions, and public health measures. This review aims to provide comprehensive insights into the health implications of air pollution and to inform strategies for lung cancer prevention, by summarising the relationship between exposure to PM2.5 and lung cancer development. We explore the different sources of PM2.5 and relevant carcinogenic mechanisms in the context of epidemiological studies on the development of lung cancer from various geographical regions worldwide.

## Introduction

According to the latest statistics released by the National Cancer Institute, lung cancer is one of the most common types of cancer worldwide and a leading cause of cancer-related deaths. It accounts for 20.8% of all cancer deaths, with 5-year survival rate of only 25.4% [1]. Men have slightly higher incidence and mortality rates for lung cancer compared to women [2], however in lung cancer in never smokers (LCINS), women have higher incidence and mortality rates than men [3]. Risk factors include exposure to direct and second-hand tobacco smoke, environmental pollutants like radon and asbestos, occupational hazards such as certain chemicals and substances, air pollution and family history of lung cancer [4, 5]. Lung cancer is classified into two main subtypes: non-small cell lung cancer (NSCLC) and small cell lung cancer [6]. Epidemiological studies have shown that exposure to air pollution is associated with an increased risk of both subtypes of lung cancer, and the strongest association is observed with adenocarcinoma in NSCLC [7–10].

Air pollution in both cities and rural areas has been linked to various respiratory problems including lung cancer [11], acute and chronic respiratory diseases, and stroke and heart diseases [12]. The combined effects of ambient air pollution and household air pollution are associated with 7 million premature deaths annually [13]. PM2.5 refers to PM with a diameter of 2.5 micrometres or smaller, this small size and weight allows PM2.5 to remain suspended in the air for extended periods. Upon inhalation, PM2.5 particles can also penetrate deep into the respiratory system and enter the bloodstream, posing local and systemic adverse health risks [14]. The World Health Organization (WHO) has issued the Air Quality Guidelines, with the latest version published in 2021 [15] stating the upper limit of recommended exposure levels for PM2.5 is 5 µg/m^3^, and for PM10 (PM 10 micrometres or smaller) is 15 µg/m^3^ [16].

The key characteristics of PM that contribute to lung cancer development include the particle number, composition, size and surface area. The composition of PM varies, with metals, organic compounds, ions and carbon being the main components [17, 18]. Heavy metals, polycyclic aromatic hydrocarbons (PAHs) and other organic constituents (e.g., endotoxins) are major contributors to the toxicity of PM [19].

Short-term exposure to high levels of PM2.5 can cause coughing, wheezing, shortness of breath, and exacerbations of asthma and other respiratory conditions [14, 20, 21]. In addition to lung cancer, long-term exposure to PM2.5 has been linked to increased risks of chronic obstructive pulmonary disease and cardiovascular disease, leading to premature death [20, 22, 23]. PM2.5 exposure increases the risk of all types of lung cancer but is most prominent for lung adenocarcinoma [23–25].

A 2023 review from the International Association for the Study of Lung Cancer (IASLC) Early Detection and Screening Committee provides an overview of air pollutants, epidemiological evidence and some mechanisms of pollution-induced carcinogenesis [23]. This review focuses on the impact of the air pollutant PM2.5 on the development of lung cancer by summarising the evidence from epidemiological studies of the relationship between different PM2.5 sources, including those produced in different geographical regions and the risk of development of lung cancer. We also discuss the likely oncogenic mechanisms of PM2.5 exposure.

## Carcinogenic effects of different sources of PM2.5

PM from different sources can carry different types and levels of toxic substances. The sources of PM can be described as either primary particles (e.g., those produced by combustion sources) or secondary particles produced by chemical reactions between gaseous precursors (e.g., sulfur dioxide, ammonia and nitrogen oxides) released from anthropogenic and natural sources [17]. Potential mediators/modulators of PM carcinogenicity include particle size and shape, surface reactivity and adhesion of various organic components [18]. Sulfates, organic compounds, PAHs, and heavy metals (such as lead, cadmium and mercury) are components of PM2.5 that are considered to be major carcinogens [26, 27]. These components increase the risk of lung cancer by causing DNA damage, inflammatory responses and oxidative stress [19, 28].

### Anthropogenic sources of PM2.5

Major anthropogenic sources of PM, include factories, combustion engines, agricultural activities, and mining. They emit a variety of PM with toxic subcomponents such as metals and chemicals. One study tracked residents from six U.S. cities and found that lowering PM2.5 levels were associated with significant reductions in death rates from heart and lung disease and lung cancer [29]. Exhaust emissions from cars, trucks, buses, and other vehicles produce PM, especially from diesel engines. Diesel exhaust fumes are classified as a Group 1 carcinogen by the International Agency for Research on Cancer [30, 31]. Traffic-Related Air Pollution (TRAP) is a significant source of ambient PM in urban areas, including PM2.5. Emissions from vehicles, especially in large cities with high traffic density, contribute substantially to air pollution [32, 33]. Vehicle emissions contain various chemical components, including PAHs, benzene, formaldehyde, and heavy metals like lead, cadmium, arsenic and nickel, many of which are known or suspected carcinogens [34, 35]. A study has shown that people living in urban areas with high levels of vehicle traffic are more likely to be exposed to elevated levels of PM2.5 and other pollutants from vehicle emissions [36], underscoring the health impacts of burning less fossil fuels and reducing emissions. Long-term exposure of TRAP PM2.5, or fine PM including diesel emissions, has been linked to an increased lung cancer risk, lung cancer mortality and cardiopulmonary disease, with significantly increased risk among truck drivers and transportation workers [37–39].

Dust and machinery emissions from construction sites are also an important source of PM2.5. Workers in the construction industry for 5 years or more have a significantly increased risk of developing lung cancer due to long-term exposure to construction PM2.5, compared to age or smoking status [40, 41]. Furthermore, a study of people near an iron foundry found that lung cancer onset was approximately 10 years earlier in men and women depending on the pollution type and distance from the foundry [42].

PM2.5 from agricultural activities are due to ammonia emissions, fertiliser use, biomass burning, and farm machinery emissions. These PM can affect human health through a number of pathways [43–45]. A study in Indonesia showed that compared with workers in other sectors, the risk of lung cancer was nearly three times higher for workers involved in crop and animal production and hunting (OR = 2.8, 95% CI = 1.11–7.02) and two times higher for workers involved in construction-related activities (OR = 1.9, 95% CI = 1.05–3.46) [46].

Among the major sources of indoor air pollution are combustion emissions from heating and cooking (e.g., burning biomass, wood, coal, and other fuels). It is worth noting that in some countries indoor fires are open fires, i.e., there is no flue to direct emissions out of the indoor space. Exposure to high levels of PM from residential heating is associated with a significantly increased risk of lung cancer [47–50]. In developing countries, there is a disproportionally risk high of lung cancer in women [47]. One study has shown that biomass PM damages mitochondria, produces reactive oxygen species, and activates p53 in human pulmonary alveolar epithelial cells, ultimately inducing apoptosis [51]. Our own studies have shown that biomass PM induces remodelling of the airway into a proliferative pre-cancerous environment [52].

### Natural sources of PM2.5

Natural sources of PM2.5 occur without direct human intervention. Wildfires generate PM2.5 that includes black carbon, organic carbon, and various carcinogenic compounds such as benzene, formaldehyde, and PAHs [53, 54]. Short-term exposure to wildfire smoke can contribute to respiratory exacerbations (e.g., in patients with asthma, chronic obstructive pulmonary disease, bronchitis and pneumonia), while exposures over a lifetime to the carcinogens in wildfire PM2.5 can increase cancer risk [53]. Acute high-level exposures pose significant health risks, for example a study from the USA shows wildland firefighters were at an increased risk of lung cancer (8–43%) and cardiovascular disease (16–30%) mortality [55–57].

PM2.5 from dust storms can carry metals and other toxic substances (e.g., silicates) depending on the soil composition [58]. In rat lung cells, exposure to desert dust extracts is associated with ROS generation, mitochondrial dysfunction, mitochondrial lipid peroxidation, and cellular antioxidant imbalance [59].

It is important to note that the composition and sources of PM can vary depending on geographic location, urbanisation, climate, and human activities. Efforts are needed to reduce PM emissions which can involve improving combustion technologies, promoting cleaner fuels, implementing dust control measures, and regulating industrial processes.

## Differences in risks of developing lung cancer due to PM2.5 geolocation

There are significant geographic differences in lung cancer incidence and mortality caused by PM2.5 exposure globally (Table 1). All studies pre-2015 have been comprehensively reviewed in the IARC monograph volume 109 [60], recent studies and notable studies pre-2015 are included in (Table 1). These differences may be influenced by a variety of factors, including local sources of air pollution, socioeconomic conditions, and public health measures [61]. There is some evidence of synergistic interaction between air pollution and cigarette smoking status and increased risk of lung cancer in different geolocations [23, 62, 63], however, this review will focus on links between air pollution and risk of lung cancer only.

**Table 1:** Epidemiological studies on the risk of air pollution exposure to lung cancer.

Reference	Study Period and Location	Sample Size	Pollutant	Outcomes
[39]	1982–1998, US	1.2 million	PM2.5	• PM2.5 associated with all-cause mortality, lung cancer mortality and cardiopulmonary mortality. • For every 10 μg/m^3^ increase in PM2.5 levels, all-cause mortality, cardiorespiratory mortality and lung cancer mortality increased by about 4%, 6% and 8%, respectively.
[64]	1982–2000, US	1.2 million	PM2.5	• PM2.5 exposure is positively associated with ischemic heart disease and lung cancer mortality.
[67]	1982–2008, US	188,699	PM2.5	• Each 10 mg/m^3^ increase in PM2.5 concentrations was associated with a 15–27% increase in lung cancer mortality. • The association between PM2.5 and lung cancer mortality were similar in men and women and across categories of attained age and education but was stronger in those with a normal body mass index and a history of chronic lung disease.
[6]	1988–2009, California US	352,053	PM2.5 PM10	• Adjusting for histology and other potential confounders, the hazard ratio associated with 1 standard deviation increases in PM10 (HR = 1.26; 95% CI: 1.25–1.28), PM2.5 (HR = 1.38; 95% CI: 1.35–1.41) for patients with localised stage at diagnosis. • Lung cancer patients with higher average ambient PM2.5 and PM10 exposures since diagnosis had shorter survival time, with the largest differences in survival for patients with early-stage non-small cell cancers (particularly adenocarcinomas).
[68]	1982–2000, US	73,711	NO_2_	• NO_2_ (a marker of traffic pollution) was associated with mortality from lung cancer.
[25]	Europe	312, 944	PM2.5 PM10	• A significant association between risk for lung cancer and PM10 (HR = 1.22; 95% CI: 1.03–1.45 per 10 μg/m^3^) and PM2.5 (HR = 1.18; 95% CI: 0.96–1.46 per 5 μg/m^3^). • The same increments of PM were associated with HRs for lung adenocarcinomas (PM10: HR = 1.51; 95% CI: 1.10–2.08 and PM2.5: HR = 1.55; 95% CI: 1.05–2.29).
[71]	2001–2010, Rome, Italy	1,265,058	PM2.5	• Strongest correlation for ischemic heart disease (HR = 1.10; 95% CI: 1.06-1.13 per 10 µg/m^3^ PM2.5), followed by cardiovascular disease and lung cancer.
[72]	2006–2010, UK	455,974	PM2.5 PM10	• Significant associations between the risk of lung cancer and PM2.5 (HR = 1.63; 95% CI: 1.33–2.01 per 5 μg/m^3^), PM10 (HR = 1.53; 95% CI: 1.20–1.96 per 10 μg/m^3^) exposure. • High air pollution exposure and high genetic risk correlate with the highest risk of lung cancer (PM2.5: HR = 1.71; 95% CI: 1.45–2.02; PM10: HR = 1.77; 95% CI: 1.50–2.10).
[70]	1986–2003, Netherlands	3355	PM2.5	• PM2.5 exposures were positively associated with all lung cancer subtypes (squamous-cell carcinoma, small-cell carcinoma, large-cell carcinoma, and adenocarcinomas)
[76]	Southern China	575, 592	PM2.5, PM10, NO_2_	• The hazard ratios for lung cancer deaths per 1 μg/m^3^ increase in PM2.5 (HR = 1.042 95% CI: 1.033–1.052), PM10 (HR = 1.032; 95% CI: 1.024–1.041), and NO_2_ (HR = 1.052; 95% CI: 1.041–1.063). • Chronic exposure to air pollution has a significant impact on lung cancer mortality rates in vulnerable populations (including the elderly).
[77]	1998–2019 Northern China	37,442	PM2.5, PM10, NO_2,_ SO_2_	• Long-term exposure to PM10 (136.5 μg/m^3^), PM2.5 (70.2 μg/m^3^), SO_2_ (113.0 μg/m^3^), and NO_2_ (39.2 μg/m^3^) was associated with an unfavourable concordance with all mortality outcomes. • A 10 μg/m^3^ increase in PM2.5 was associated with higher mortality from lung cancer (HR = 1.14; 95% CI: 1.05–1.23).
[81]	2000–2015, Taiwan	174,431	PM2.5	• For never smokers, the risk of developing lung cancer (HR = 1.32; 95% CI: 1.12–1.56) and dying from lung cancer-related causes (HR = 1.28; 95% CI: 1.01–1.63) rises significantly with every 10 μg/m^3^ increment of PM2.5 exposure, but not for smokers.
[82]	1995–2015, Taiwan	371,084	PM2.5	• PM2.5 levels affect the adenocarcinoma lung cancer incidence and survival. • Five-year survival rates for never-smokers, those with EGFR wild-type genes, and female patients with adenocarcinoma lung cancer were 12.6% in North Taiwan and 4.5% in South Taiwan (HR = 0.79; 95% CI: 0.70–0.90).
[80]	2010-2014 Japan	/	PM2.5, CO, NO_2,_ SO_2_	• Increases PM2.5, NO_2_, SO_2_, and CO concentrations were associated with 2.65% (95% CIs: 0.96%–4.37%), 4.28% (95% CIs: 2.24%–6.36%), 3.35% (95% CIs: 1.03%–5.73%), and 4.60% (95% CIs: 2.19%–7.05%) increased risk of lung cancer mortality, respectively. • Associations were strongest in the elderly and men.

### The United States of America

A study by the American Cancer Society revealed chronic exposure to combustion-related PM2.5 as a significant environmental risk factor for lung cancer mortality, with each 10 μg/m³ increase in PM2.5 concentration correlating with ~8% increase in lung cancer mortality [39]. A follow-up study over seven years confirmed a significant positive correlation between increasing PM2.5 concentrations and lung cancer mortality. It highlighted that two covariates, high school level education rate and median household income, had the greatest impact on this association [64–66]. Moreover, a comprehensive nationwide 26-year study demonstrated that even in the absence of tobacco exposure, long-term exposure to PM2.5 significantly elevated the risk of lung cancer, which was consistent across genders, age groups, and education levels, but notably stronger in individuals with normal body mass index and a history of chronic lung disease [67]. Focusing on survival rates, a study from California found that lung cancer patients with higher mean ambient PM2.5 and PM10 exposures had shorter survival periods after diagnosis, with the most significant differences among patients with early-stage NSCLC, particularly adenocarcinomas [6]. Another California-based study analysed the correlation between PM2.5, O_3_, NO_2_ exposure and various diseases, including lung cancer [68]. Results indicated that areas with higher PM2.5 concentrations had higher lung cancer mortality rates [68]. A longitudinal study of Canadian women further supported these findings, reporting the risk of lung cancer increased by 34% for every 10 μg/m³ rise in PM2.5 levels (HR: 1.34; 95% CI = 1.10, 1.65) [69]. These studies consistently show that long-term exposure to PM2.5 is a significant risk factor for lung cancer mortality in The United States of Americas.

### Europe

A meta-analysis of 17 cohort studies from 9 European countries showed a significant association between lung cancer risk and PM2.5 exposure, with the hazard ratio of 1.18 (95%CI:0.96–1.46) per 5 μg/m^3^ increase [25]. Increases in PM2.5 are highly associated with lung adenocarcinoma [25]. Regarding the incidence of subtypes of lung cancer, the Dutch Diet and Cancer Cohort Study found that long-term exposure to high levels of TRAP PM2.5 was positively associated with the risk of all lung cancer subtypes [70]. Long-term exposure to NO_2_ and PM2.5 is associated with increased lung cancer mortality in Rome, Italy [71], further supporting the relationship in a European context. Additionally, in a prospective study using the UK Biobank, long-term exposure to ambient PM2.5 is associated with increased risk of lung cancer in people with high genetic risk (e.g. defined by 18 single nucleotide polymorphisms) [72].

### Asia

In the following section it is important to note that associations between PM levels and lung cancer incidence occur in all Asian countries. China and Japan are listed as examples for high and low air pollution.

China is one of the most polluted countries in the world [73], due to rapid urbanisation and industrialisation over the past few decades [74]. A study estimated that 23.9% of lung cancer deaths in China were associated with PM2.5 exposure in 2015 [75]. A recent study based on a large cohort in southern China found that long-term exposure to polluted air (PM2.5, PM10 and NO_2_) had a significant impact on lung cancer mortality, especially in elderly and people who exercise regularly [76]. For people in northern China, long-term exposure to high concentrations of PM10, PM2.5, SO_2_, and NO_2_ is associated with increased lung cancer mortality [77]. Importantly, the largest contributor is PM2.5. A 10 μg/m^3^ increase in PM2.5 was associated with a hazard ratio of 1.14 for lung cancer mortality (CI:1.05–1.23) [77]. Another analysis of 72 national cancer registries in China showed that annual lung cancer incidence rates in men highly correlated with PM2.5 levels [78]. A nation-wide study in China showed that lung cancer deaths due to PM2.5 exposure were increased by 76,000 in the period from 2004 to 2012 [79]. These studies consistently show that long-term exposure to higher PM2.5 concentrations is significantly associated with lung cancer mortality in a dose-dependent manner and with significant regional differences.

A study in Osaka, Japan, evaluated the association between short-term air pollution exposure and lung cancer mortality. Air pollutants (PM2.5, CO, NO_2,_ and SO_2_) were associated with an increased risk of death from lung cancer, and the association was most pronounced in the elderly and in men [80].

Taken together, there is growing evidence of the link between long-term exposure to ambient PM2.5 and lung cancer mortality or morbidity from cohort studies around the world [25, 70, 71, 81, 82]. In addition, trends in ambient PM2.5 exposure varied markedly from country to country, with lower ambient PM2.5 exposure in developed countries and higher ambient PM2.5 exposure in less-developed and moderately developed countries, which may be related to local socioeconomics, air quality policies, and lung cancer prevention measures [83]. Longitudinal studies of children in high TRAP/PM2.5 areas will be important to assess the increased risk of developing lung cancer in adulthood in different geolocations. Furthermore, inclusion of the characterisation of PM2.5 composition in different geographical locations may reveal specific components of PM2.5 that correlate with the observed differences in epidemiology.

Furthermore, a study from patients in East Asia showed specific chromosome regions containing oncogene or tumour suppressor genes (such as TP53, ERBB2, MYC and APC) enriched for carcinogen signatures (nitroamine-like, nitro-PAH, radiation, alkylating agents, PAHs) [84]. This study suggests that components of PM2.5 (that are known carcinogens) may be involved in mutagenesis and further studies assessing geolocation-specific PM2.5 on mutational profiles in different populations is warranted and critical for understanding how environmental exposures cause cancer.

## Mechanisms of PM2.5 induced carcinogenesis

**Fig. 1:**
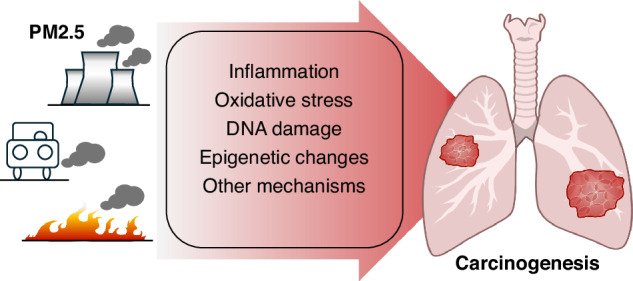
Mechanisms of PM2.5-induced carcinogenesis.

PM2.5 exposure plays an important role in the development of lung cancer, possibly through mechanisms such as inflammation, oxidative stress, DNA damage and epigenetic changes (Fig. 1). The in vitro and in vivo models of PM and lung cancer are summarised in Table 2 and Table 3, respectively.

**Table 2:** Experimental studies on PM exposure and lung cancer in vitro.

Carcinogens	Cell Type	Treatment (dose and timing)	Effects	Reference
Ambient PM2.5	• Human bronchial epithelial cells (HBE cells) • HEK293T cells	100 μg/ml PM Up to 48 h	• PM up to 48 h increases TRAF6, CXCL2, IL-8 levels.	[87]
Ambient PM2.5 (Collected at Kowloon Tong of Hong Kong)	• Lung cancer cell line: • NCI-H23 Lung normal bronchial epithelial cells: • Bet1A	5 μg/ml PM2.5 Up to 28 days	• PM2.5 significantly enhanced cell proliferation, migration and invasion of both cell lines • Overexpression of 15-LOX1/15-LOX2 prevented PM2.5 changes in migration and vimentin levels.	[88]
Ambient PM2.5	• HBE cells	100 or 500 μg/ml PM2.5 48–72 h per passage Continued for 30 passages	• Long-term exposure to PM2.5 increases proliferation, migration and invasion. • Knockdown of ATP citrate lyase reversed PM-induced EMT, migration and invasion.	[105]
Ambient PM2.5 (Beijing and Shijiazhuang)	• Human lung adenocarcinoma cell lines: • H1299 • HCC827 • A549 Human bronchial epithelial cells: • BEAS-2B cells	10 μg/mL PM2.5 14 days	• PM2.5 promotes migration and invasion in HCC827 and BEAS-2b cells. • PM2.5 increases EMT and the expression of cancer stem cell-related genes in A549 and H1299 cells.	[106]
Traffic-originated PM2.5 organic component (tPo)	• H1299 • A549	5 μg/mL and 2 μg/mL tPo 10 passages	• 5 μg/ml and 2 μg/ml tPo treatment increased proliferation and migration of A549 and H1299 cells. • Chronic tPo treatment upregulated loc107985872-notch signalling in A549 and H1299 cells.	[131]
PM2.5	• A549	16 μg/cm^2^ PM2.5 12, 24 or 48 h	• PM2.5 exposure increases loc146880 (lncRNA) expression in A549 cells. • PM2.5-induced loc146880 increases autophagy and promotes cell migration, invasion and EMT.	[132]
PM2.5	• A549 cells	Pretreatment: 10, 20, 30 μM biochanin A for 2 h. 190 μg/mL PM2.5 24, 48, and 72 h.	• PM2.5 induces EMT in A549 cells. • Biochanin A pretreatment reverses PM-2.5-induced EMT *via* PI3K/Akt signalling pathway.	[107]
PM2.5	• A549 • PC9 • BEAS-2B	100, 200, 400 μg/ml PM2.5 24–48 h.	• PM2.5 increases glycolysis in A549, PC9 and BEAS-2B cells. • PM2.5 exposure increases the expression of DLAT in a dose-response manner in BEAS-2B cells.	[134]
PM2.5	• A549 • H1975 cells	50 μg/mL PM2.5 90 days	• Chronic exposure to PM2.5 significantly increased the ability of H1975 and A549 cells to undergo adherent, non-dependent growth. • In H1975 cells, PM2.5 increased pEGFR, AhR and TMPRSS2-IL18 pathways.	[133]
PM2.5 PAHs	• HBE cells	Stable transfection of CYP1A1 into HBE cells. Up to 100 μg/mL PM 4 weeks.	• PM increased proliferation and genes associated with malignant transformation in HBE-CYP1A1 cells in a dose-dependent manner.	[139]
PM2.5 PAHs	• HBE cells	Up to 100 μg/mL PM	• miRNA profiles are significantly changed in the PM2.5-induced malignant transformation cells. • PM increases miR-200 family • miR-200a-3p targets Tensin-3 to increase migration.	[130]

**Table 3:** Experimental studies on PM exposure and lung cancer in vivo.

Carcinogens	Animal	Treatment	Effects	Reference
Ambient PM2.5	Male C57BL/6 J, *lc3b*^−/−^ and *Becn1*^+/−^ mice	100 μg PM (in 50 μl of saline) intranasal instillation 5 times. Luciferase-labelled LLC or B16-F10 cells (tail vein injection). 5 × 10^5^ 4T1 cells (subcutaneous injection).	• PM exposure promotes lung pre-metastatic niche formation (seeding of B15-F10 cells or LLC cells), and lung metastasis (4T1 cells in lung) compared to saline-treated controls.	[87]
Ambient PM2.5 (Kowloon Tong of Hong Kong)	Nude mice (5–week-old female)	Xenograft mouse model NCI-H23 lung cancer cells (5 × 10^6^) were treated with 5 μg/ml PM_2.5_ or 10 μM NNK for 28 days prior to subcutaneous injection into mice.	• Tumour volume increased in mice implanted with PM2.5- or NNK-treated NCI-H23 cells compared to controls.	[88]
PM derived from stainless steel (SS) welding	A/J Mice (5–6 weeks)	A/J mice were IP injected with MCA (initiator), or corn oil (vehicle) then 1 week later exposed to chromium-containing gas metal arc (GMA)-SS welding PM (340 or 680 μg) or PBS (vehicle; sham) by pharyngeal aspiration once a week for 5 weeks. Mice were culled 30 weeks post-initiation.	• GMA-SS increased tumour multiplicity and gross tumour number at low and high doses. • Low dose GMA-SS group also had presence of inflammatory cell infiltrates in histology sections.	[140]
Ambient particulate matter (PM2.5)	Nude mice (Female)	Xenograft mouse model Control-HBE cells and ACLY knockdown (KD) HBE cells treated with 100 μg/mL PM2.5 (30 passages). 1×10^7^ cells were mixed with 0.5 ml DMEM medium and injected subcutaneously on the ventral flanks of nude mice. Mice were culled at 21 days.	• PM2.5 increased tumour number and volume. ACLY KD + PM2.5 had less and smaller tumours than control groups.	[105]
Ambient PM2.5 (Beijing and Shijiazhuang)	NOD/SCID mice (Male)	Patient-derived xenograft model. Human tumour samples were divided into small pieces (1–2 mm diameter) and transplanted into the NOD/SCID mice subcutaneously. On days 1, 8, 15, 22, 29, 36 and 43 after engraftment, mice were administered 50 μL PM2.5 (1 mg/mL or 5 mg/mL) by intranasal instillation.	• Chronic exposure to PM2.5 promotes the tumorigenesis and metastasis (kidney).	[106]
PM2.5	4-week-old male Sprague Dawley rats	PM2.5 (1.8, 5.4, 16.2 mg/kg body weight) administered intratracheally every 3 days for 24 days. Controls rats received saline.	• PM2.5 exposure increased L-lactate, pyruvate, DLAT in lung tissues compared to control rats.	[134]
PM2.5 PAHs	BALB nude mice	1 × 10^7^ HBE-1A1 cells treated with up to 100 μg/mL organic extracts (PM) injected subcutaneously in nude mice. Mice were culled at 16 weeks post injection.	• Similar tumorigenicity in PM-treated HBE-1A1 cells injected nude mice from 3 geographic locations.	[139]

### Inflammation and oxidative stress

Several clinical studies have shown that both acute and chronic inflammation are associated with an increased risk of various malignancies [85–88]. Oxidative damage also plays an important role in the development of many cancers [89, 90]. Inflammation causes oxidative damage to cellular components through the formation of reactive oxygen species (ROS) and reactive nitrogen species [91–93].

The general roles of tumour-associated macrophages (TAMs), neutrophils (TANs) and MDSCs have been comprehensively reviewed elsewhere [94–98]. Notably, human studies show that increased oxidative stress and inflammation in the lungs of people exposed to cigarette smoke or PM2.5 are associated with increased lung disease and mortality rates [89]. Many of the substances in PM2.5 can affect immune cells and initiate an inflammatory response in the lungs [99, 100], including the organic compounds PAHs. PM2.5 can increase the recruitment of immune cells, such as macrophages, neutrophils and T cells [99], and also impair the function of cytotoxic CD8+ T cells and NK cells [101] – which under homeostatic conditions are critical for immune surveillance and tumour elimination.

These PM2.5 compounds stimulate respiratory epithelial cells and macrophages to release inflammatory mediators, such as IL-1β, IL-6, IL-8, and TNF-α, thereby activating the NF-κB signalling pathway, leading to increased proliferation and migration, and epithelial-mesenchymal transition (EMT), which all increase the risk of cancer [102–107]. Furthermore, fine PM alters macrophage immunometabolism and promotes defective alveolar macrophage responses, resulting in increased tumourigenesis in the lung [108].

The heavy metals (e.g., lead, cadmium, and chromium) in PM2.5 increase ROS production and induce the release of inflammatory mediators from lung cells which further promotes the development of lung cancer [89, 102, 109].

An in vitro study using a human bronchial epithelial cell line demonstrated that PM exposure induces neutrophil chemotaxis and initiates ROS-mediated autophagy in the alveolar epithelium to promote lung cancer metastasis [87]. Degradation of the E3 ubiquitin ligase Tripartite Motif Containing 37 (TRIM37) protects TNF Receptor-Associated Factor 6 (TRAF6) from proteasomal degradation in the lung epithelium, which in turn promotes the production of NFκB-dependent chemokines to recruit neutrophils, creating the conditions for the formation of a pre-metastatic microenvironment for lung cancer [87]. Another study found that acute exposure to 1-NPs (a PAH) increased pro-inflammatory cytokines and chemokines (such as IL-1β, IL-6, TNF and keratinocyte chemoattractant [KC]) and activated PI3K/Akt signalling when administered intratracheally to wild type mice and also in human A549 cells [110]. PM2.5 exposure can also change the formation of the lung tissue matrix, which impedes T cell movement and function, and leads to accelerated lung tumourigenesis [101].

### DNA damage

DNA damage can increase the risk of lung cancer due to genomic instability, mutations, and dysregulated repair mechanisms [111]. Oncogenic driver mutations are present in over 50% of lung cancer, including mutations in EGFR, KRAS, BRAF, PI3K, MEK-1, HER2, MET, ALK and RET and inactivation of tumour suppressor genes (such as P53, PTEN, LKB-1) (reviewed in [112]).

PM2.5 is capable of causing DNA strand breaks and mutations through inflammation combined with oxidative stress [113]. The mechanisms primarily include the activation of oxidative stress-related signalling pathways (such as NF-κB and MAPK), oncogenes, inactivation of tumour suppressor genes, mutations in repair genes, and microsatellite instability [114–116]. Concurrently, it can activate oncogenes (such as c-Myc, Ras, and ERK) that promote abnormal cell proliferation while inactivating tumour suppressor genes (such as p53 and RB1) [117]. Additionally, PM2.5 exposure may result in mutations or inactivation of DNA repair genes like BRCA1/2, ATM and ATR, which impairs the ability to repair damaged DNA further exacerbating genomic instability [118–120].

Different components of PM2.5 may also contribute to lung cancer related DNA damage. PAHs can bind to DNA and trigger oxidative stress [102]. Heavy metals (e.g., lead, cadmium, chromium) directly interact with DNA to cause DNA strand breaks and apoptosis [103]. The DNA repair enzyme OGG1 is important in these processes [116].

A landmark 2023 study identified an association between PM2.5 exposure and incidence of EGFR-driven lung cancer [121]. Importantly, this study showed that in early tumourigenesis PM2.5 exposure in mice with pre-existing EGFR mutation promotes transcriptional reprogramming of AT2 cells to a state that is reminiscent of a progenitor cell [121].

Therefore, PM2.5 induces DNA damage through oxidative stress, inflammation, and impaired repair mechanisms, leading to genomic instability and impaired repair mechanisms, which significantly to cause increase the risk of lung cancer. Also, PM2.5 exposure can initiate early tumorigenesis in lung cells with pre-existing mutations.

### Epigenetic changes

Epigenetic mechanisms play a key role in the pathogenesis of PM2.5-induced lung cancer. Epigenetic alterations can be divided into three main categories: DNA methylation, histone modifications, and non-coding RNA regulation.

#### DNA methylation

DNA hypermethylation or hypomethylation profiles represent silenced or activated target genes, respectively, and thus can repress or increase gene expression. Normal methylation is perturbed in lung cancer, for example 11 CpG islands are methylated in more than 80% of squamous cell carcinomas [122]. Thus, DNA methylation markers are expected to be used a biomarkers for early lung cancer detection, such as hypermethylation of the tumour suppressor gene p16 [123]. PM2.5 exposure can cause alterations in DNA methylation, especially in the promoter regions of oncogenes [124].

It has been found that continuous exposure of BEAS-2B cells to PM2.5 can methylate the p53 promoter, leading to p53 inactivation, and the ROS/Akt signalling pathway is also involved in methylation [125]. A recent PM2.5-induced methylome analysis of BEAS-2B cells identified 66 differentially expressed genes, mostly associated with tumour suppression, that have been linked to lung disease, particularly lung cancer [126]. Biomass smoke extract stimulates primary human lung fibroblasts, leading to the upregulation of IL-6 and IL-8 release, and the mechanism by which biomass smoke exposure increases lung inflammation can be targeted and inhibited through the p38 MAP kinase pathway [127]. These studies provide a basis for further exploration of the association between PM2.5-induced DNA methylation and lung cancer.

#### Histone modifications

Histone modifications can affect histone-DNA interactions and alter chromatin structure and function, which can lead to genomic instability and altered gene expression. PM is known to effect histone modifications, for example PM-induced histone H4 acetylation leads to exposure increased IL-8 release from A549 cells by increasing histone H4 acetylation [128]. Furthermore, a small cohort study found histone 3 lysine 27 acetylation was related to PM2.5 exposure levels [129]. Whilst epigenetic changes are known to promote the development of cancer, no study has definitively shown that PM-induced lung cancer is medicated via histone modifications.

#### Non-coding RNAs

MicroRNAs (miRNAs) are RNAs 20–22 nucleotides in length, which are involved in the regulation of post-transcriptional gene expression and RNA silencing. They achieve this by binding to complementary sequences on target mRNAs, leading to either the degradation of the mRNAs or inhibiting the translation of proteins. However, how miRNAs contribute to the pathogenesis of PM2.5-induced lung cancer remains unclear. A study used a variety of primary benign lung epithelial cell lines and lung cancer cell lines and constructed a human-derived xenograft model [106]. It was found that PM2.5 exposure (once per week for 6 weeks) promoted tumorigenesis and metastasis in lung adenocarcinoma in the xenograft, as well as the migration and invasion of lung adenocarcinoma cell lines [106], though downregulation of miRNAs (miR30a, miR125a, miR200a, miR200c, miR221, Let7C) and increased gene expression of fibrotic and inflammatory factors (including N-Cadherin, Fibronectin-1, Vimentin, Snail1, Slug, Zeb1, Zeb2, CD44, Abcg2, CD133) [106]. Another study modelled PM2.5-induced malignant transformation in vitro, where the miR-200 family, especially miR-200a-3p, was involved in the development of lung cancer induced by PM2.5 exposure, and miR-200a-3p promoted cell migration by directly inhibiting tensin 3 expression [130].

Long-stranded non-coding RNAs (lncRNAs) are a class of RNAs over 200 nucleotides in length that do not code for proteins. They can be involved in the regulation of gene expression and cellular function by binding to and inhibiting the function of miRNA. Chronic exposure to PM2.5 organic fraction tPo upregulated the expression of loc107985872 via notch1 signalling pathways, promoted lung adenocarcinoma cell invasion and migration and EMT [131]. PM2.5 exposure also upregulates lncRNA loc146880 via increased ROS production. Loc146880 can uncontrollably increase autophagy and promote cell migration, invasion and EMT in A549 (lung carcinoma) cells [132].

### Additional mechanisms

Signal transduction pathways play a crucial role in cancer development and progression. These pathways regulate cell growth, division, apoptosis and differentiation. Once these pathways are dysregulated, it may lead to uncontrolled proliferation, inhibition of apoptosis, invasion and metastasis of cancer cells. Chronic exposure to PM2.5 promotes lung cancer by enhancing the TMPRSS2-IL18 pathway through activation of epidermal growth factor receptor and aryl hydrocarbon receptor [133]. PM2.5 can also induce spindle-like changes in cell morphology, which gives cancer cells their ability to migrate and invade, via activation of the PI3K/Akt signalling pathway, and induction of EMT in lung cancer cells [107]. PM2.5 has also been shown to increase glycolysis through increased dihydrolipoyl transacetylase expression (a key component of the pyruvate dehydrogenase complex) [134]. Furthermore, PM2.5 also increases hypoxia-inducible factor (HIF-1) and downstream VEGF to promote angiogenesis in cell and animal model systems [135].

## Conclusion

Air pollution is associated with several health conditions, including lung cancer [19, 136], emphasising the importance of addressing air pollution as a public health priority. Implementing measures to reduce PM2.5 levels, such as enforcing stricter emission standards for vehicles and industrial processes, promoting clean energy sources, and encouraging sustainable urban planning, can significantly mitigate the health risks associated with air pollution, including lung cancer [137, 138]. Further studies are required to better understand the mechanisms of action and assess novel treatment approaches to reduced air pollution-associated lung cancer.
